# Population Genetic Structure and Origins of Native Hawaiians in the Multiethnic Cohort Study

**DOI:** 10.1371/journal.pone.0047881

**Published:** 2012-11-07

**Authors:** Sung K. Kim, Christopher R. Gignoux, Jeffrey D. Wall, Annette Lum-Jones, Hansong Wang, Christopher A. Haiman, Gary K. Chen, Brian E. Henderson, Laurence N. Kolonel, Loic Le Marchand, Daniel O. Stram, Richa Saxena, Iona Cheng

**Affiliations:** 1 Institute for Human Genetics, University of California San Francisco, San Francisco, California, United States of America; 2 Sequenom, Incorporated, San Diego, California, United States of America; 3 Epidemiology Program, University of Hawai'i Cancer Center, Honolulu, Hawaii, United States of America; 4 Department of Preventive Medicine, Norris Comprehensive Cancer Center, Keck School of Medicine, University of Southern California, Los Angeles, California, United States of America; 5 Program in Medical and Population Genetics, Broad Institute, Cambridge, Massachusetts, United States of America; 6 Center for Human Genetics Research, Massachusetts General Hospital, Boston, Massachusetts, United States of America; Vanderbilt University, United States of America

## Abstract

The population genetic structure of Native Hawaiians has yet to be comprehensively studied, and the ancestral origins of Polynesians remain in question. In this study, we utilized high-resolution genome-wide SNP data and mitochondrial genomes of 148 and 160 Native Hawaiians, respectively, to characterize their population structure of the nuclear and mitochondrial genomes, ancestral origins, and population expansion. Native Hawaiians, who self-reported full Native Hawaiian heritage, demonstrated 78% Native Hawaiian, 11.5% European, and 7.8% Asian ancestry with 99% belonging to the B4 mitochondrial haplogroup. The estimated proportions of Native Hawaiian ancestry for those who reported mixed ancestry (i.e. 75% and 50% Native Hawaiian heritage) were found to be consistent with their self-reported heritage. A significant proportion of Melanesian ancestry (mean = 32%) was estimated in 100% self-reported Native Hawaiians in an ADMIXTURE analysis of Asian, Melanesian, and Native Hawaiian populations of K = 2, where K denotes the number of ancestral populations. This notable proportion of Melanesian admixture supports the “Slow-Boat” model of migration of ancestral Polynesian populations from East Asia to the Pacific Islands. In addition, approximately 1,300 years ago a single, strong expansion of the Native Hawaiian population was estimated. By providing important insight into the underlying population structure of Native Hawaiians, this study lays the foundation for future genetic association studies of this U.S. minority population.

## Introduction

Population structure and genetic ancestry of Native Hawaiians, a racial/ethnic minority group within the U.S., have been understudied. Previous reports of the genetic admixture of Native Hawaiians have been largely based on ancestry informative markers (AIMs), which can be limited in their resolution in providing accurate estimates of the genetic contribution of ancestral populations [1], [2]. Studying these patterns of population structure is imperative for genetic association testing in which the confounding effects of population stratification can lead to false positive associations [3], [4]. In addition, individuals with mixed ancestry such as Native Hawaiians can be used to map susceptibility loci for complex traits via admixture mapping. This is a powerful approach for localizing risk loci of traits that have a higher prevalence in one ancestral population than another [5]–[11]. Such an approach has yet to be applied to Native Hawaiians due to the lack of a comprehensive admixture map for this group. Future admixture mapping studies in Native Hawaiians may be informative for chronic diseases such as cardiovascular disease, diabetes, and breast cancer for which Native Hawaiians demonstrate a higher incidence than other Asian and European populations [12]–[16].

Native Hawaiians have a long and complicated demography, dating back to the earliest settlement of Hawai'i. Archeological and paleoecological evidence suggest that the Hawaiian archipelago was initially settled by Polynesian settlers between 300 AD and 800 AD [17]. Ancestral Polynesians are widely believed to originate from East Asia [18], [19]. However, as they migrated across the Pacific (remote Oceania), their degree of admixture with the indigenous Melanesian population is unclear. Two leading yet polar demographic models have been proposed. The first model, the “Express Train” hypothesis, suggests that the migration of ancestral Polynesians across the Pacific was rapid with limited or no contact with the local Melanesian population. Evidence in support of this theory includes linguistics [18]–[21] and mitochondrial data [22]–[25]. The second model, the “Slow Boat” hypothesis, suggests that ancestral Polynesians intermixed significantly with Melanesians prior to populating the Pacific Islands. Evidence in support of this hypothesis includes archaeological evidence [26] and Y chromosome data [27]–[30]. Previous genome-wide surveys [31]–[33] that addressed the ancestral origins of Polynesians have yielded conflicting results over these two hypotheses with the majority of studies focusing on microsatellite (short-tandem repeat) markers [31], [32]. These discrepancies may be due to the use of microsatellite markers, which have inherently high mutation rates and a mutation model that is difficult to model [34]–[36]. More recently, Wollstein et al. [33] used genome-wide SNP data as a more precise and fine-resolution approach to understand the demographic history of Polynesians, estimating ∼13% Melanesian ancestry with admixture occurring 3,000 years ago.

The population size of Native Hawaiians during the 17^th^–18^th^ century ranged from 250,000–800,000 individuals [17]. Tragically, due to an epidemic of new diseases brought by visiting sailors and immigrant settlers, the Native Hawaiian population underwent a drastic population bottleneck, such that the 1900 census reported only ∼40,000 Native Hawaiians. Most recently, the Native Hawaiian population has experienced nearly a five-fold increase in size with the U.S. 2000 census estimating over 200,000 individuals [37] (recognizing self-reporting preferences may influence this estimate, possibly leading to an overestimation in reporting of Native Hawaiian ancestry due to cultural preferences). Several diverse ethnic groups migrated to Hawai'i, following Captain James Cook's discovery of the Hawai'i islands in 1778, primarily due to the development of a sugar production industry and the resultant need for a large cadre of plantation workers. Historical records indicate that the first reported Chinese immigrants migrated to Hawai'i on merchant ships in 1788 [17], [38] with as many as 56,000 additional Chinese immigrants migrating as contract workers between 1852 and 1899 [39]. In addition, over 200,000 Japanese also migrated to Hawai'i from 1885 through 1924 [40]. Overall, hundreds of thousands of individuals from all around the world, including the Philippines, Portugal, and Puerto Rico, migrated to Hawai'i via labor recruitment efforts [17]. Native Hawaiians have often intermarried with persons of other racial/ethnic groups, and these marriages have led to significant interbreeding among the diverse populations that migrated to Hawai'i [17]. The drastic reduction in population size of Native Hawaiians after the arrival of Europeans and their increase in numbers after annexation by the U.S. suggest a rich and complicated demographic history similar to other admixed populations within the U.S., such as Latinos and African Americans [17] [41]–[43].

In this study, we present a comprehensive fine-scale analysis of the genome-wide estimates of population structure and admixture in Native Hawaiians, using high-resolution genotype data from both the nuclear and mitochondrial genomes. Our genome-wide SNP data of the nuclear genome gives us an opportunity to examine the degree of population structure within Native Hawaiians. In addition, we test two competing hypotheses on the ancestral origins of Polynesians as represented by Native Hawaiians. Furthermore, our sequencing data of the mitochondrial genome allows for characterizing the patterns of mitochondrial DNA (mtDNA) diversity in Native Hawaiians as well as estimating the effective population size of Native Hawaiians as seen through time.

## Results

### Genome-wide data

**Figure 1:**
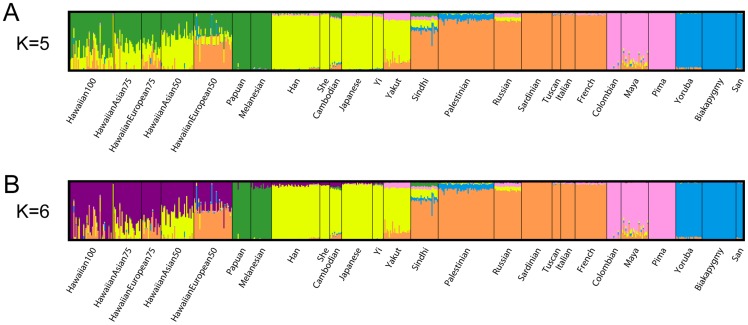
ADMIXTURE clustering of Native Hawaiians for K = 5 (A) and K = 6 (B). **Figures 1A and 1B** illustrate the clustering of Native Hawaiians and HGDP samples based on GWAS data. Each vertical bar represents an individual's proportion of K ancestral clusters (i.e. color) as estimated by ADMIXTURE.

**Figure 2:**
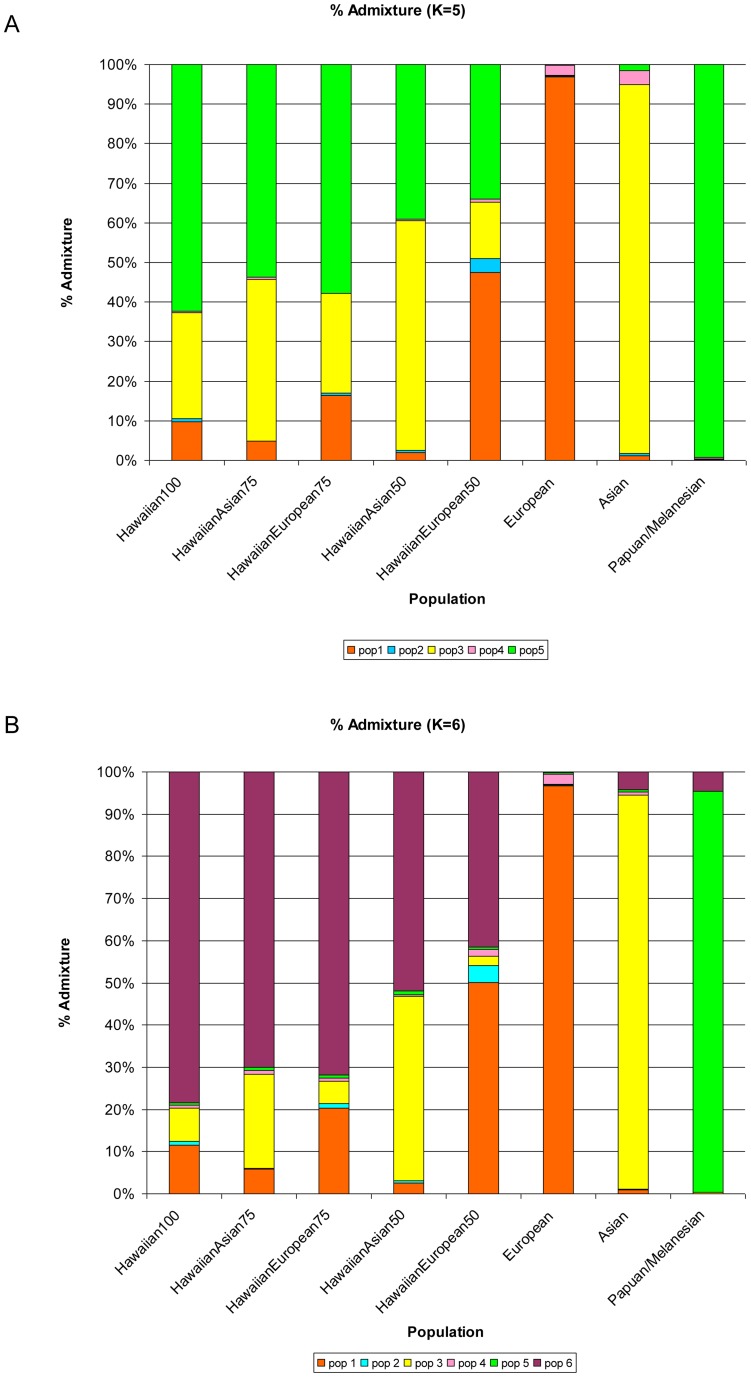
Average of each ancestral clustering estimated by ADMIXTURE for K = 5 and K = 6. Figures 2A and 2B illustrate the mean values for each proportion of ancestry based on GWAS data. Pop1-6 are the ancestral populations representing the European, African, East Asian, American, Oceanian, and Hawaiian populations, respectively.

We applied ADMIXTURE on genome-wide SNP data to finely estimate the degree of admixture in Native Hawaiians. We analyzed 148 Native Hawaiians by using a merged dataset of 114,112 SNPs and 466 additional individuals from HGDP (see Methods). Genome-wide SNP analysis at K>7 identified continental substructure (such as the differentiation between our representative African populations) whereas K<5 identified major continental groups (data not shown as they provide little information in regards to the ancestral composition of Native Hawaiians). Figures 1A and 1B show ADMIXTURE's estimate of each individual's genome-wide proportion of ancestry when K = 5 and K = 6. K = 5 and K = 6 were selected as they provided the first clear differentiation of Native Hawaiians relative to the major ethnic populations within our study. Each color was assigned to represent one of the K putative ancestral populations by determining which reference population group contained the largest ancestral component. We find, as Figure 2A illustrates, that for K = 5, on average, 37.7% (the sum of all non-green components of ancestry) of the Native Hawaiian genomes of those who reported themselves and both their parents as being solely Native Hawaiian originated from a population outside Oceania. ADMIXTURE analysis at K = 6 suggests that these 100% self-reported Native Hawaiians have an average of 78% of their genomes classified as Native Hawaiian ancestry (min = 0.019, max = 0.99, median = 0.87, std. dev. = 0.25) with 11.5% and 7.8% classified as European and Asian ancestry, respectively (Figure 2B).

As expected, individuals with founders originating from non-Hawaiian populations typically have higher levels of admixture when compared to individuals with full Native Hawaiian lineage. For example, at K = 5, individuals with either parent belonging to Asian or European descent, ADMIXTURE estimated 58.0% and 47.5% of their genomes to contain Asian and European ancestry, respectively (Figure 2A). At K = 6, we find Native Hawaiians, whose grandparent (as assumed based on reporting of parental background) was Asian (European) to contain 22.3% (20.3%) of their genome to be of Asian (European) ancestry (Figure 2B).

**Figure 3:**
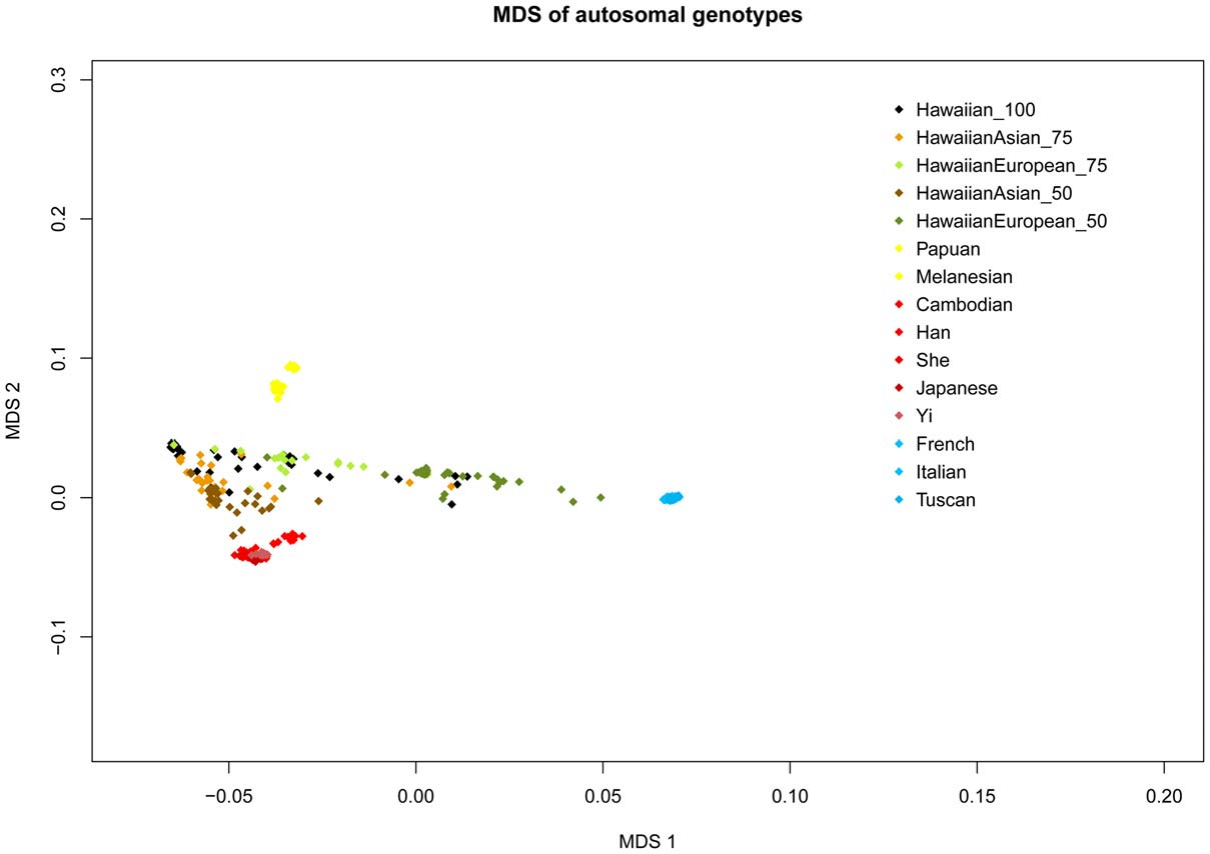
Multi-Dimensional Scaling analysis of GWAS data. HGDP representative samples with European, East Asian and Oceanian ancestry and are plotted against Native Hawaiians with various degrees of self-reported ancestry in MDS dimensions.

We performed a MDS analysis of the genome-wide SNP data by using the MDS routine in PLINK to generate a graphical view of the genomic distance between each individual. Figure 3 illustrates the clustering of individuals belonging to select populations that represent three major groups: Europeans, Asians and Oceania. Consistent with ADMIXTURE estimates of ancestry, we find that Native Hawaiians typically cluster between all three divergent populations. Moreover, admixed Native Hawaiians with European (Asian) ancestry tended to cluster more towards the representative European (Asian) populations. These findings suggest a great level of variation among Native Hawaiians today, quite possibly due to historical and ongoing admixture events, and warrant careful estimation of admixture with more individuals.

### Origins of Native Hawaiians

**Figure 4:**
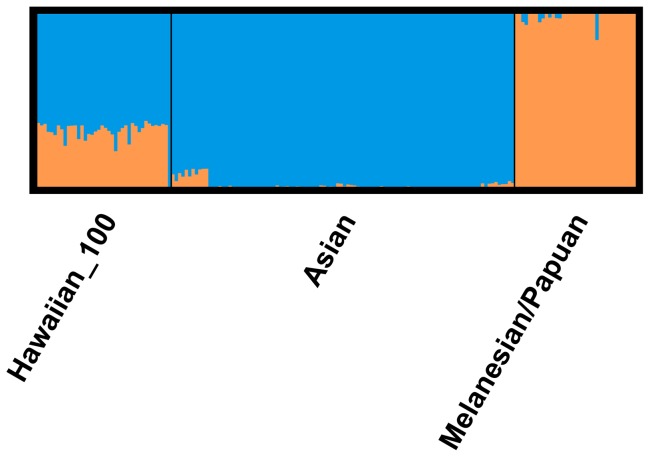
Supervised ADMIXTURE results for K = 2 using Native Hawaiians, East Asian and Oceanian populations.

The “Express Train” and the “Slow Boat” models of Polynesian migration are expected to have uniquely distinct genetic signatures on present day genomes of Native Hawaiians. Under the “Express Train” model, the proportion of admixture in Native Hawaiians of Melanesian and Asian ancestry is expected to be near zero, whereas under the “Slow Boat” model, the proportion of admixture is expected to be substantially greater than zero. To test these two models, we conducted a supervised ADMIXTURE analysis using Papuan and Melanesians as one source population of Polynesians and Han Chinese, She, Cambodian, Japanese, Yakut, and Yi as surrogates for the second source population of Taiwanese aborigines [18], [19]. Importantly, we did not fix ancestry for the Melanesians or Asians and therefore allowed for admixture within either ancestral groups–thus, mitigating bias by earlier admixture processes and allowing for accurate clusters of ancestry membership. We set K = 2 and estimated in 40 100% Native Hawaiians an average of 32% and 68% of their genomes to be derived from Melanesian and Asian origins, respectively (Figure 4). This notable proportion of Melanesian admixture (32%) among Native Hawaiians, substantially greater than zero, lends support of the “Slow Boat” model of ancestral origins.

### Mitochondrial DNA data

The Polynesian motif is comprised of a 9-bp COII/tRNA^Lys^ intergenic deletion relative to the revised Cambridge Reference Sequence (rCRS) for the mitochondrial genome and accounts for >90% of Polynesian mtDNA [24]. Lum et al. [44] identified the Polynesian motif in ∼95% of their Native Hawaiian samples, consistent with archeological and linguistic evidence of Polynesian origins [45]. In the 160 Native Hawaiian mitochondrial genomes for this study (100% self-reported Native Hawaiian ancestry), we find 80, 19, and 60 carry the B4a1a1, B4a1a1a1 and B4a1a1a3 motif, respectively. Furthermore, we identified one individual, who carried 7 of the 9 diagnostic markers for the Q1 motif (Table S5). Across all 160 Native Hawaiians, we identified 14 mtSNPs to have a minor allele frequency >1% (Table 1), of which 6 polymorphisms have not been previously identified by PhyloTree [46] to be a haplogroup identifying marker.

**Table 1:** Native Hawaiian specific mitochondrial SNP with minimum allele frequency >1%.

mtDNA position	Reference allele	Native Hawaiian allele	Minor Allele Frequency	Haplogroup Marker
841	A	G	0.093	
1185	C	T	0.377	B4a1a1a3
1692	A	G	0.325	M27c, G2b2a, U6a7a
3398	T	C	0.038	M65b, I2a1
3639	A	G	0.013	
4314	T	C	0.013	P10
6905	A	G	0.129	B4a1a1a1, M5c2
8538	T	C	0.013	
9145	G	A	0.019	
10451	T	C	0.036	
12245	T	C	0.019	R3
14022	A	G	0.994	B4a1a1
15746	A	G	0.994	B4a1a, G3a, N8
15776	A	G	0.013	

Figure S1 depicts the MDS analysis with select samples of mitochondrial haplogroups. We selected haplogroups P and B as they are commonly observed in Polynesian populations [47]–[49]. To provide a broad spectrum of ancestral haplogroups for comparison to our Native Hawaiian mitochondrial haplogroups, we included lineages from haplogroups H, J, K, L, M, D and G. Overall, we find consistent patterns with the known genealogy of our reference panel of mitochondrial sequences. The majority of our Native Hawaiians appears to cluster amongst themselves with some overlap with haplogroup B, a haplogroup found across East Asia, the Pacific, and the Americas. In light of the drastic isolation, genetic drift, population bottleneck and subsequent growth that occurred in the Hawaiian Islands after settlement, we see a clear signature of Polynesian lineages as an outlying cluster.

**Figure 5:**
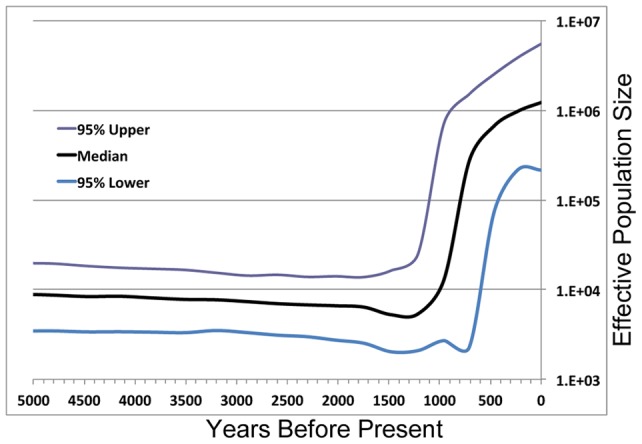
BEAST analysis of 160 Native Hawaiian mitochondrial genomes. All default values were used to generate the effective population size as a function of time with 95% confidence intervals.

The Bayesian Skyline analysis in Figure 5 shows a single, strong expansion beginning approximately 1,300 years before present (median estimate). From that point on, the Native Hawaiian effective population size expands two orders of magnitude within 1,000 years. The beginning of the expansion correlates strongly with archaeological records for the initial settlement of the Hawai'i islands. It is worth noting that if we used the phylogenetic mutation rate, our estimates would have been far too early (roughly 4,000 Years Before Present).

## Discussion

In this study, we integrated nuclear and mitochondrial genomic data from Native Hawaiians with HGDP data to provide the first comprehensive survey of the three components of their demography–patterns of population structure, genetic origins, and population expansion over time.

Our genome-wide SNP data provides a unique view into the genetic architecture and the degree of admixture in Native Hawaiians. We find patterns of admixture when examining genome-wide SNP data in 100% self-reported Native Hawaiians such that on average 11.5% and 7.8% of their genomes contained European and Asian ancestry, respectively. In addition, we detected admixture to be consistent with the expected ancestral proportions based on varying degrees of self-reported Native Hawaiian ancestry. Given the high number of markers available in genome-wide array data, we are well-powered to determine fine-scale differences in cluster membership [50]–[52]; thus, we do not expect this measured admixture to be biased by ancient shared ancestry such as seen in Asian and Oceanic populations.

Overall, our results are roughly comparable to the report by Wang et al. [53] that estimated 74% Hawaiian ancestry based on 300 AIMs that were tested in Multiethnic Cohort subjects, who reported Native Hawaiian heritage. We recognize the possibility of errors in self-reported ancestry and our data set is hampered by the lack of male individuals that would allow for investigation of sex-biased demography, a genomic signal detected in modern human populations [54], particularly in admixed populations, such as Puerto Ricans [55] and Polynesian populations [48]. To address these limitations and further refine the ancestral contribution of European and Asian ancestry in Native Hawaiians, future studies should develop specific AIMs for Native Hawaiians as well as include larger study populations of Native Hawaiian men and women with high-density genome-wide SNP data.

Recently, Kayser et al. [32] surveyed the nuclear genome with 377 microsatellite markers in 47 Pacific Islanders and identified 79% Asian and 21% Melanesian proportions of ancestry for Polynesians. These findings, along with evidence of excess Melanesian origin of the Y chromosome [27]–[30] and excess Asian origin of the mtDNA [22]–[25], suggest a sex-biased demography of admixture between ancestral Polynesians and indigenous Melanesians consistent with the “Slow Boat” hypothesis [48]. Contrary to Kayser et al. [32], Friedlaender et al. [31] also conducted a genome-wide survey with 687 microsatellites in 952 individuals from 41 Pacific populations and concluded that their data were supportive for the “Express-Train” hypothesis. Both studies are limited in genome-wide interpretations of the origins of Polynesians due to their use of microsatellites. Microsatellites are known to have higher mutation rates and a mutation model that is difficult to model, which are less reliable for studies geared toward demographic inference [34]–[36]. In addition, previous studies on the Y chromosome and mtDNA are single-locus analyses and can provide only a limited view of past demographic events.

Our study of 488,961 autosomal SNPs of 40 100% Native Hawaiians provides the largest study to date that tests two divergent hypotheses regarding the ancestral migration patterns of Polynesians, specifically Native Hawaiians. Based on our supervised ADMIXTURE analyses, we estimated on average, 32% of the Native Hawaiian genome originates from Melanesian ancestry. This notable proportion of Melanasian admixture, substantially greater than zero, which would be expected under the “Express Train” model, conversely supports the “Slow Boat” hypothesis of ancestral origins. Although our conclusions are consistent with a recent genome-wide SNP study of 25 Polynesians by Wollstein et al [33], who examined highland Papuan groups, our estimation of Melanesian ancestry may have overestimated the true admixture proportions given that we based our analysis on ancestral populations (namely the coastal/island Melanesian groups found within the HGDP panel) that have historical Asian admixture via the Austronesian expansion. Furthermore, given the complex demographic history of Native Hawaiians, our current analyses cannot differentiate between long-range versus short range admixture events. While an analysis of variance of ancestry may provide insights into migration rates and estimate the time of the admixture events [56] such analysis may be confounded by overfit models.

Lastly, we merged 160 100% Native Hawaiian mitochondrial sequences with 544 a priori selected mitochondrial sequences available in the public domain to query the patterns of polymorphisms. The importance of understanding the genetic patterns of the mitochondria as demonstrated by Biffi et al. [57] is that the mtDNA can serve as a powerful tool to complement genome-wide data in assessing the confounding factors of population stratification in genome-wide association studies. Consistent with previous reports [44], we find that 99% of Native Hawaiians in our sample contain the Polynesian motif. We further show a clustering of our Native Hawaiian mitochondrial genomes towards the general B haplogroup, which previously has been identified among Melanesian, Micronesian, and Polynesian populations [58]. Given our observation of moderate levels of historical admixture with Europeans and Asians, the observed near fixation of the Polynesian motif in full Native Hawaiians suggests the occurrence of sex biased demography with an excess of European/Asian males and Native Hawaiian females in the ancestral population of Native Hawaiians.

The Skyline analysis demonstrates a single, strong expansion after the initial settlement of the islands. In addition, the single mode of expansion is concordant with a single wave of settlers, rather than a two-wave process that has been put forward in the literature [17], [59], [60]. Although Polynesians contact with Westerners in the past few hundred years has resulted in a marked decrease in census population size, the effective population size was large enough so findings concerning the earlier period of expansion should not have been largely affected by such an event. The median population today appears to be overestimated, however, the credible interval is consistent with the current census population. At any rate Skyline analyses (consistent with other coalescent approaches) will have more uncertainty with extremely recent estimates due to the paucity of very recent substitution events [61], [62].

Recent next-generation sequencing approaches have demonstrated the issues of demographic inference with mitochondrial data due to biased sampling approaches [63]. However, in our analysis this is not a concern as we selected 160 Native Hawaiians for sequencing from a population-based cohort and all samples were used in the Skyline analysis. The fact that they nearly all exhibit the Polynesian motif is consistent with Native Hawaiians being at the terminus of a serial expansion and limits our resolution to more ancient events. Therefore, the recent expansion signal observed in this analysis would not be due to bias in sampling but represents the colonization of the Hawaiian Islands.

It is feasible that this pattern of explosive growth may preserve an excess of rare variants across the entire genome of Native Hawaiians in comparison to populations that have not experienced such a recent rise in effective population size. Similar patterns have been detected in large candidate gene resequencing studies in diverse populations [64]. Future large-scale sequencing studies of Native Hawaiians should take into account that the allele frequency spectrum of the Polynesian component of this population would be notably shifted towards rare variants, even though they are at a terminus of a long serial founder effect model across the Pacific Ocean [65].

Given the dynamic and complex history of Native Hawaiians, along with the large influx of various ethnic groups into the islands over the past 200 years, it is possible that the observed admixture signals in individuals, who reported full Native Hawaiian heritage, are residuals of historical admixture events. Although our dataset is modest in comparison to studies of predominantly European populations that incorporate thousands of individuals genotyped on genome-wide SNP arrays [66], our study does, nevertheless, notably contribute to our basic understanding of the population genetics of Native Hawaiians, an understudied minority group. In addition, our estimation of the explosive growth of the Native Hawaiian population, likely leading to an increase in rare variants, has important implications for future sequencing studies of complex diseases in this population. The information gained here lays the foundation for future genetic association studies of Native Hawaiians by providing insights into the necessary corrections for errors incurred due to confounding factors of population stratification. Moreover, our findings suggest that mapping by admixed linkage disequilibrium may be a powerful tool in elucidating the genetic etiology of complex traits in Native Hawaiians.

## Materials and Methods

### Study Subjects

The Multiethnic Cohort Study is a large population-based prospective study of more than 215,000 men and women from Hawaii and California (mainly Los Angeles County). The cohort is composed predominantly of individuals from the following five racial/ethnic groups: African Americans, Native Hawaiians, Japanese, Latinos, and European Americans. Participants between the ages of 45 and 75 years were recruited from March 1993 through May 1996. Participants were asked to self-report their race/ethnicity and that of each of their parents, which included the option to report a mixed racial/ethnic background. Further details about this cohort are provided elsewhere [67].

We utilized high-resolution genome-wide SNP data and mitochondrial genomes of 148 and 160 Native Hawaiians, respectively (discussed below). In total, we had 192 individuals, who self-identified as solely Native Hawaiian ancestry and reported only Native Hawaiian ancestry for each parent (herein we refer as 100% self-reported Native Hawaiian). Thirty and 35 individuals identified one parent belonging to either an Asian or European-ancestry descent group, respectively, which we classified as 50% Native Hawaiian. The remaining 25 and 18 individuals comprised of those who reported one of their parents heritage as Asian and/or European, which we classified as 75% Native Hawaiian. We classified Asian heritage as those who reported themselves as Japanese, Korean, Chinese, and/or Filipino (see Table S1 and Table S2) for descriptions of each individual as well as the available data). This study was approved by the institutional review boards at the University of Hawaii and the University of Southern California. Written informed consent was obtained from all participants.

### Genome-wide data and analysis

To gain a high-resolution estimate of genome-wide admixture in Native Hawaiians, we utilized genome-wide data for 148 Native Hawaiians (of whom 40 self-reported as 100% Native Hawaiians). Genotyping was conducted using the Illumina Infinium 660W bead array at the University of Southern California as part of an on-going genome-wide association study of breast cancer (Native Hawaiian cases/controls = 79/69). Of the 561,490 SNP probes, 72,529 were excluded due to a low minor allele frequency (MAF<0.01), poor completion rate (<0.95), or poor concordance (<0.99) across 75 intended replicate samples. We selected a subset of individuals from the Human Genetic Diversity Panel (HGDP; n = 466) [68]-[70] (Table S1) that included samples from two Oceanic populations (Papuans and Melanesians), European ancestry (Utah residents from the CEPH population, Sardinians, Tuscans, French, Italians, and Russians), Africa (Yoruba, Biaka Pygmies, and San), South-Central Asia/Middle East (Sindhi and Palestinians), Central/South America (Mayans, Colombians, and Pima), and Asia (Han Chinese, She, Cambodians, Japanese, Yakut, and Yi). Individuals from Asian and Oceania populations were selected to represent potential source populations for Native Hawaiian while the remaining groups were chosen as contrast for population structure analyses. After initial quality control filtering, data was available for 670,372 and 488,961 SNPs for the HGDP and Native Hawaiian samples, respectively. Using PLINK [71], we further filtered the joint set of autosomal SNPs for genotyping call rates >99.5%, minor allele frequencies >5%, HWE (P>0.001), and SNPs in linkage disequilibrium (r^2^<0.4),. In total, 114,112 SNPs and 614 individuals were used by ADMIXTURE [72] to estimate each individual's proportion of ancestry for varying values of K ancestral clusters in which K ranged from 2 to 8. We utilized PLINK [71] to conduct a multi-dimensional scaling (MDS) analysis of the genome-wide data to generate a graphical view of the genomic distance between individuals.

### Mitochondrial genome data and analysis

To identify the mitochondrial haplogroup diversity of Native Hawaiians and estimate their population expansion, we sequenced the 16.5 kb mitochondrial genome in 160 100% self-reported Native Hawaiians, using the Affymetrix GeneChip Human Mitochondrial Resequencing Array 2.0. This chip provides an economical and highly accurate method for assessing variation in the mitochondrial coding regions [73]. Mitochondrial DNA was amplified in two PCR reactions using 100 ng of genomic DNA. Fragmentation, labeling, and chip hybridization was conducted according to manufacturer's instruction. Sequence analysis was conducted using the Affymetrix GeneChip Sequence Analysis Software (GSEQ) v4.1 software. GSEQ uses an objective statistical framework, based on the ABACUS algorithm, to assign base calls to each position according to quality criteria (default settings were used). The Revised Cambridge Reference Sequence (rCRS) was used as the reference sequence. The average base call rate of the mitochondrial genome was 90.6%. We validated the use of the Affymetrix Mitochondrial Resequencing Array by independently sequencing 2% replicate samples using the Pyrosequencing technology. We observed a 100% concordant rate in sequence calls.

6,686 full mitochondrial sequences were downloaded from NCBI (as of February 2010) using the following command: Homo [Organism] AND gene_in_mitochondrion [PROP] AND 14000∶19000 [SLEN] NOT pseudogene [All Fields]. Using build 8 of PhyloTree [46], we extracted 2,621 full mitochondrial sequences with previously annotated mitochondrial haplogroup assignments. Each mitochondrial sequence was then independently aligned to rCRS via KALIGN version 2.0.3 [74], and all variant positions were annotated relative to rCRS base positions. For each of the 2,781 total mitochondrial sequences (including the 160 Native Hawaiians we sequenced), we extracted all aligned base information, excluding the hypervariable region, and identified the diagnostic mitochondrial SNP (mtSNP) from MITOMAP [75] for a given mitochondrial haplogroup as well as any mtSNP with a minor allele frequency greater than 2%. MDS was then performed on 252 mtSNPs and 704 mitochondrial sequences (160 of which were Native Hawaiian) by using the subroutine *cmdscale* within R software (Tables S3 and S4). We chose MDS, rather than Principal Component Analysis, as it can handle potential missing data without imputation. Detailed mitochondrial haplogroup categorization was determined for each Native Hawaiian mtDNA sequence by the presence or absence of the B4 sub-lineage haplogroup mutations found in build 8 of PhyloTree [46].

We further used the 160 Native Hawaiian mitochondrial genomes to estimate demographic parameters of the Hawaiian population, using the Bayesian Skyline process in the BEAST software package [76]. This Bayesian algorithm estimates effective population size (N_e_) through time via coalescent theory by using a nonparametric Markov Chain Monte Carlo. The Bayesian Skyline has been shown to be a highly accurate method of detecting fluctuations in population sizes in humans [77]–[79]. In particular, rapid expansions, such as island colonization, can be captured by this method.

In order to run Bayesian Skylines, we first isolated the coding region of each mitochondrial genome and aligned it to rCRS by using the coding region coordinates described in MITOMAP. These regions were analyzed using default settings of the BEAST software. As the observed mutation rate in mtDNA is known to decrease with a higher number of coalescent events [80], we kept the mutational clock in generation-based units in BEAST and applied a post-run correction for the mutation rate at each generation. This method has been shown to be more accurate and consistent with archaeological dating methods than assuming a constant mutation rate through time [79]. There is still a debate in the field as to why this mutation rate slowdown occurs [81], [82], however, when estimating the timing of demographic events in the recent past, the empirical estimator calibrated from archaeological evidence [76] and a model incorporating purifying selection [77] give qualitatively similar results.

## Supporting Information

Figure S1 **MDS analysis of mitochondrial haplogroup.**
(TIF)

Table S1 **Sample information for GWAS and mitochondrial sequence data.**
(XLS)

Table S2 **Summary counts of subjects used for AIMS, GWAS, and mtDNA analysist.**
(XLS)

Table S3 **Individuals used for MDS mtDNA sequence analysis and corresponding haplogroup.**
(XLS)

Table S4 **Corresponding mtDNA position numbers used for mtDNA sequence analysis and rCRS allele.**
(XLS)

Table S5 **Nine diagnostic markers for mtDNA haplogroup Q1 and allelic states for individual 52 and rCRS.**
(XLS)
