# Supplementary figures and images for: Population Genetic Structure and Origins of Native Hawaiians in the Multiethnic Cohort Study
Source: PLoS One. 2012 Nov 7;7(11):e47881. doi: 10.1371/journal.pone.0047881 (PMC3492381; doi:10.1371/journal.pone.0047881)

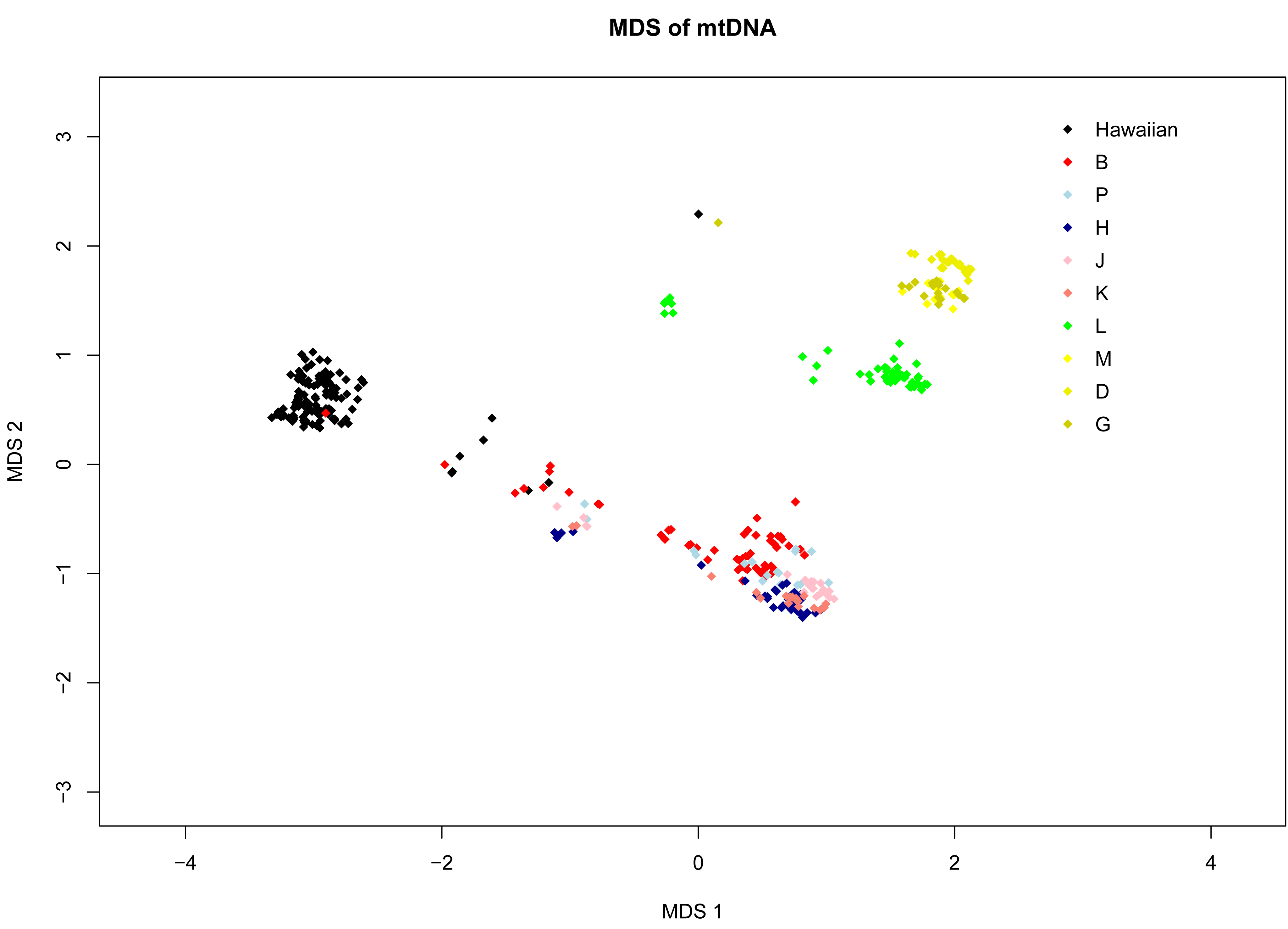

Supplement: Figure S1 — MDS analysis of mitochondrial haplogroup. (TIF) [file pone.0047881.s001.tif]
